# Factors affecting treatment outcome in patients with idiopathic nonspecific interstitial pneumonia: a nationwide cohort study

**DOI:** 10.1186/s12931-017-0686-7

**Published:** 2017-12-06

**Authors:** Sang Hoon Lee, Moo Suk Park, Song Yee Kim, Dong Soon Kim, Young Whan Kim, Man Pyo Chung, Soo Taek Uh, Choon Sik Park, Sung Woo Park, Sung Hwan Jeong, Yong Bum Park, Hong Lyeol Lee, Jong Wook Shin, Eun Joo Lee, Jin Hwa Lee, Yangin Jegal, Hyun Kyung Lee, Yong Hyun Kim, Jin Woo Song, Jong Sun Park

**Affiliations:** 1 Department of Internal Medicine, Division of Pulmonary and Critical Care Medicine, Seoul National University Bundang Hospital, 82 Gumi-ro, 173 Beon-gil, Bundang-gu, Seongnam-si, Gyeonggi-do 463-707 Republic of Korea; 2 Yonsei University College of Medicine, 50-1 Yonsei-ro, Seodaemun-gu, Seoul, 120-752 South Korea; 3 Department of Internal Medicine, Division of Pulmonology, Severance Hospital, Institute of Chest Diseases, Yonsei University College of Medicine, 50-1 Yonsei-ro, Seodaemun-gu, Seoul, 120-752 South Korea; 4 Division of Pulmonary and Critical Care Medicine, University of Ulsan College of Medicine, Asan Medical Center, Seoul, South Korea; 5 Department of Internal Medicine and Lung Institute, Division of Pulmonary and Critical Care Medicine, Seoul National University College of Medicine, Seoul, South Korea; 6 Division of Pulmonary and Critical Care Medicine, Samsung Medical Center, Sungkyunkwan University School of Medicine, Seoul, South Korea; 7 Department of Internal Medicine, Division of Allergy and Respiratory Medicine, Soonchunhyang University Seoul Hospital, Seoul, South Korea; 8 Department of Internal Medicine, Division of Allergy and Respiratory Medicine, Soonchunhyang University Bucheon Hospital, Bucheon, South Korea; 9 Department of Internal Medicine, Division of Pulmonology, Gachon University Gil Medical Center, Incheon, South Korea; 10 Department of Internal Medicine, Division of Pulmonary, Allergy & Critical Care Medicine, Kangdong Sacred Heart Hospital, Hallym University, Seoul, South Korea; 11 Department of Internal Medicine, Pulmonary Division, Inha University Hospital, Incheon, South Korea; 12 Department of Internal medicine, Division of Pulmonary Medicine, Chung Ang University College of Medicine, Seoul, South Korea; 13 Department of Internal Medicine, Division of Respiratory and Critical Care Medicine, Korea University Anam Hospital, Korea University College of Medicine, Seoul, South Korea; 14 Department of Internal Medicine, Ewha Medical Research Institute, Ewha Womans University School of Medicine, Seoul, South Korea; 15 Department of Internal Medicine, Division of Pulmonary Medicine, Ulsan University Hospital, University of Ulsan College of Medicine, Ulsan, South Korea; 16 Department of Internal Medicine, Division of Critical Care and Pulmonary Medicine, Inje University Busan Paik Hospital, Busan, South Korea; 17 Department of Internal Medicine, Division of Allergy and Pulmonology, Bucheon St. Mary’s Hospital, The Catholic University of Korea School of Medicine, Bucheon, South Korea

**Keywords:** Non-specific interstitial pneumonia, Treatment, Pulmonary lung function

## Abstract

**Background:**

The effects of corticosteroid-based therapy in patients with idiopathic nonspecific interstitial pneumonia (iNSIP), and factors affecting treatment outcome, are not fully understood. We aimed to investigate the long-term treatment response and factors affecting the treatment outcome in iNSIP patients from a multi-center study in Korea.

**Methods:**

The Korean interstitial lung disease (ILD) Study Group surveyed ILD patients from 2003 to 2007. Patients were divided into two groups to compare the treatment response: response group (forced vital capacity (FVC) improves ≥10% after 1 year) and non-response group (FVC <10%). Factors affecting treatment response were evaluated by multivariate logistic regression analysis.

**Results:**

A total of 261 patients with iNSIP were enrolled, and 95 patients were followed-up for more than 1 year. Corticosteroid treatment was performed in 86 patients. The treatment group showed a significant improvement in lung function after 1-year: FVC, 10.0%; forced expiratory volume (FEV_1_), 9.8%; diffusing capacity of the lung for carbon monoxide (DLco), 8.4% (*p* < 0.001). Sero-negative anti-nuclear antibody (ANA) was significantly related with lung function improvement. Sero-positivity ANA was significantly lower in the response group (*p* = 0.013), compared to that in the non-response group. A shorter duration of respiratory symptoms at diagnosis was significantly associated with a good response to treatment (*p* = 0.018).

**Conclusion:**

Treatment with corticosteroids and/or immunosuppressants improved lung function in iNSIP patients, which was more pronounced in sero-negative ANA and shorter symptom duration patients. These findings suggest that early treatment should be considered in iNSIP patients, even in an early disease stage.

**Electronic supplementary material:**

The online version of this article (10.1186/s12931-017-0686-7) contains supplementary material, which is available to authorized users.

## Background

Non-specific interstitial pneumonia (NSIP) is a type of interstitial idiopathic interstitial pneumonia (IIP) mainly affecting female non-smokers aged 40–60 years. Although more rigorous studies are needed, the prevalence of idiopathic NSIP (iNSIP) is estimated to be between 1 and 9 in 100,000 [1, 2]. NSIP can present as idiopathic or is associated with secondary conditions, such as connective tissue disease (CTD), human immunodeficiency virus infection, IgG4-related disease, bone marrow transplant, or toxin/drug-related conditions [2–4]. In addition, NSIP with connective tissue disease has recently been re-classified as interstitial pneumonia with autoimmune disease [5].

Although the natural course of iNSIP is not yet known, previous studies showed that the prognosis of NSIP is favorable when compared with idiopathic pulmonary fibrosis (IPF) [6–8]. Corticosteroid and immunosuppressive agents (including azathioprine, cyclophosphamide, cyclosporine, and mycophenolate mofetil) are widely used and thought to be beneficial for patients with NSIP [3, 9, 10]. However, changes to pulmonary function are not fully understood in both untreated and treated NSIP patients, especially in patients with low severity NSIP. Additionally, previous studies have addressed the risk factors and medical conditions associated with mortality rate, relapse, and progression of the disease, but the degree of response and factors affecting treatment have not been well studied [10, 11].

The Korean Interstitial Lung Disease (ILD) Research Group performed a nationwide survey to investigate the characteristics of patients with ILD, including iNSIP. In the present multicenter, nationwide study, we aimed to investigate the effect of treatment and the factors affecting the treatment outcome in patients with surgically proven-iNSIP.

## Methods

### Study population

**Fig. 1:**
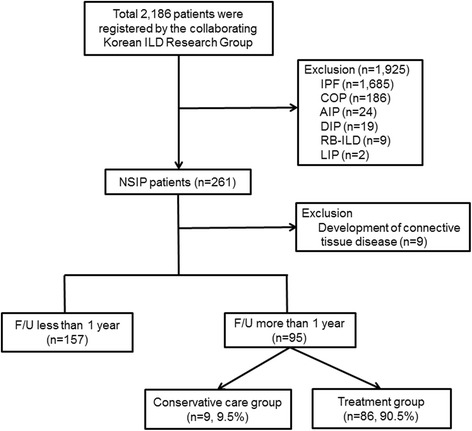
Flow chart of the study population. Initially, 2186 patients with interstitial lung disease (ILD) were enrolled by the Korean study group between January 1, 2003, and December 31, 2007. A total of 261 surgically diagnosed patients with non-specific interstitial pneumonia (NSIP) were analyzed in this study; 1925 patients with a different diagnosis other than NSIP were excluded from this study. One hundred and fifty seven patients were followed-up within 1 year, and 95 patients were followed-up after >1 year. Of these 95 patients, nine were in the conservative care group, and 86 in the treatment group. The treatment group was defined as those prescribed corticosteroid and/or immunosuppressant therapy, and conservative care group was defined as those who were only prescribed medication for symptom control. IPF: idiopathic pulmonary fibrosis, COP: cryptogenic organizing pneumonia, AIP: acute interstitial pneumonia, DIP: desquamative interstitial pneumonia, RB-ILD: respiratory bronchiolitis-associated interstitial lung disease, LIP: lymphocytic interstitial pneumonia

Figure 1 shows the patient-flow chart. In total, 2186 idiopathic interstitial pneumonias (IIP) patients were registered by the Korean ILD Research Group, which includes pulmonologists from 54 University hospitals across the country with more than 500 beds starting 2006, from January 1, 2003, to December 31, 2007. Patients with a history of using medication that could provoke ILD (e.g., amiodarone or cytotoxic agent), and had a collagen-vascular disease were excluded from the study. In addition, patients with granulomatous diffuse parenchymal lung disease (e.g., sarcoidosis) or a rare form of ILD (e.g., lymphangioleiomyomatosis or pulmonary Langerhans cell histocytosis) were initially excluded from the study. NSIP was diagnosed based on the American Thoracic Society/European Respiratory Society (ATS/ERS) 2002 guidelines via a multidisciplinary approach by a pulmonologist, a chest specific radiologist, and pathologists [12]. Patients diagnosed with IIP other than NSIP, including acute interstitial pneumonia, cryptogenic organizing pneumonia, desquamative interstitial pneumonia, lymphocytic interstitial pneumonia, nonspecific interstitial pneumonia, and respiratory bronchiolitis-associated interstitial lung disease, were excluded. Patients diagnosed clinically without a surgical lung biopsy were excluded. Additionally, patients for whom definitive diagnoses could not be made at each hospital, were reviewed by the Scientific Committee of the Korean Academy of Tuberculosis and Respiratory Diseases. Among the 261 patients with NSIP, those who developed a new CTD (*n* = 9) were excluded. Patients with hypersensitivity pneumonitis (HP), as indicated by the patient’s history, clinical symptoms, and serologic test results, were also excluded from the study. Finally, 252 patients with iNSIP were analyzed in this study; 157 patients were followed-up within 1 year, and 95 patients were followed-up after >1 year. Clinical (age, gender, smoking status, smoking amount, respiratory symptom, comorbidity, and outcome), physiological (pulmonary function test [PFT]), and laboratory (arterial blood gas analysis, C-reactive protein, anti-nuclear antibody [ANA], and rheumatoid factor) findings were retrospectively investigated. All patients’ data were recorded in a web-based registry (www.ild.or.kr).

The 95 patients who were followed-up after >1 year were divided into two groups: a no treatment group (*n* = 9) and a treatment group (*n* = 86). Patients who were prescribed corticosteroid or immunosuppressive agents were defined as “treatment group”. The mean duration of treatment was 11.8 ± 8.3 months. The no treatment group patients were either only prescribed medication for symptom control or no medication at all.

To determine the effect of the treatment, the treatment group was further divided into two sub-groups. The response group was defined as patients with a change of ≥10% between the initial predicted forced vital capacity (FVC) (%) and the 1-year follow-up predicted FVC (%). The non-response group was defined as patients with a change of <10% between the initial predicted FVC (%) and the 1-year follow-up predicted FVC (%).

### Statistical analysis

Continuous variables were expressed as the mean ± standard deviation (SD) or median with interquartile range and compared by Student’s t-test or a Mann Whitney U-test according to the distribution of patients. Categorical variables were presented as frequency (n) and percentage (%), and were analyzed by Fisher’s exact test or Pearson’s chi-square test. A Wilcoxon signed rank test or Paired t-test were conducted to compare the effect of treatment (initial PFT results vs 1-year follow-up results). A repeated measures ANOVA was conducted to compare the change in lung function between the response group and non-response group at 1 year. A logistic regression model was used to investigate the factors affecting the treatment outcome. An adjusted *p* value <0.05 was considered to indicate significance. SPSS™ Version 22.0 (SPSS, Chicago, Illinois, USA), was used for all statistics analysis.

## Results

Among 252 patients with iNSIP, 95 patients who were followed-up for over 1 year were analyzed to evaluate treatment response in this study. Table 1 shows the characteristics of the patients with iNSIP. The mean age was 57.1 ± 10.7 years, and females (65.1%) were predominant. The mean follow-up duration was 21.6 ± 16.6 months among all patients, and 32.1 ± 13.9 months in patients followed-up after >1 year. Overall lung function was slightly decreased compared to normal; FVC (%) was 71.8 ± 18.4, forced expiratory volume (FEV_1_) (%) was 79.8 ± 20.9, and diffusing capacity of the lung for carbon monoxide (DL_CO_) (%) was 64.9 ± 21.3. Most of the patients were not smokers. Dyspnea (77.8%) and cough (72.6%) were the most common respiratory symptoms.

**Table 1:** Characteristics of the study population

	Total patients (*n* = 261)	Patients followed up more than 1 year (*n* = 95)
Age (year)	57.1 ± 10.7	56.2 ± 10.3
F: M	170 (65.1): 91 (34.9)	64 (67.4): 31 (32.6)
^*^Follow-up duration (month)	21.6 ± 16.6	32.1 ± 13.9
Pulmonary function test		
FVC (%)	71.8 ± 18.4	72.5 ± 19.3
FEV_1_ (%)	79.8 ± 20.9	81.1 ± 21.2
DLco (%)	64.9 ± 21.3	64.7 ± 22.5
^*^Resting PaO_2_ mm Hg	81.6 ± 17.3	86.7 ± 14.8
Smoking status		
Never smoker	169/241 (70.1)	68/92 (73.9)
Ex-smoker	38/241 (15.8)	16/92 (17.4)
current smoker	34/241 (14.1)	8/92 (8.7)
^*^Smoking amount (Pys)	29.3 ± 16.8	29.0 ± 15.2
Initial symptom		
Dyspnea of exertion	179/230 (77.8)	64/80 (80.0)
Cough	159/219 (72.6)	55/81 (67.9)
Sputum	69/186 (37.1)	28/71 (39.4)
^*^Duration of symptom (month)	6.2 ± 10.2	7.1 ± 12.0
^*^CRP (mg/dL)	2.52 ± 6.21	1.56 ± 3.68
ANA (positive)	58/185 (31.4)	18/59 (30.5)
RF (positive)	28/173 (16.2)	11/59 (18.6)
Outcome		
Alive	157/261 (60.2)	73/95 (76.8)
Death	26/261 (10.0)	3/95 (3.2)
Follow-up loss	78/261 (29.9)	19/95 (20.0)

Note: Values in parentheses are percentages

*F:M* female:male, *FVC*, forced vital capacity, *% pred* percentage of the predicted value, *FEV*_*1*_ forced expiratory volume, *DL*_*CO*_ diffusing capacity of the lung for carbon monoxide, *PaO*_*2*_ arterial oxygen tension, *CRP* C-reactive protein, *ANA* antinuclear antibody, *RF* rheumatoid factor

^*^Follow-up duration, smoking amount, duration of symptoms, and CRP showed a non-normal distribution in all patients

^*^Resting PaO_2_ mm Hg, duration of symptom, and CRP showed a non-normal distribution in patients followed up for more than 1 year

Clinical, physiologic, and laboratory data for the no treatment group and treatment group are shown in Table 2. More than 95% of patients were treated with steroid (Additional file 1: Table S1). In the treatment group, the median age was lower than that in the treatment group, although the difference was not significant (*p* = 0.061). Gender, follow-up duration, PFT results, smoking, initial respiratory symptoms, laboratory results, and comorbidities did not differ significantly between the groups (Table 2 and Additional file 1: Table S2). The change in lung function between the initial visit and the 1-year follow-up was investigated (Additional file 1: Table S3). In the no treatment group, although the FVC (%), FEV_1_ (%), and DL_CO_ (%) were increased 1 year after diagnosis, compared to the initial assessment, these changes in PFT were not significant (*p* = 0.276, *p* = 0.400, and *p* = 0.489, respectively). However, in the treatment group, these values were all significantly improved after 1 year (*p* < 0.001, all).

**Table 2:** Characteristics according to treatment in patients followed-up for more than one year

	No treatment group (n = 9)	Treatment group (n = 86)	*P*-value
Age (year)	63.0 (57.5, 66.0)	55.0 (48.0, 64.3)	0.061
F: M	5 (55.6): 4 (44.4)	59 (68.6): 27 (31.4)	0.467
Follow-up duration (month)	23.0 (20.5, 43.0)	30.5 (20.8, 42.0)	0.412
Pulmonary function test			
FVC (%)	83.0 (63.5, 91.0)	69.5 (58.0, 87.0)	0.291
FEV_1_ (%)	100.0 (75.0, 104.5)	80.0 (64.0, 95.0)	0.111
DLco (%)	70.5 (61.3, 79.3)	48.0 (61.0, 80.0)	0.165
^*^Resting PaO_2_ mm Hg	85.0 (76.3, 91.0)	89.0 (77.5, 98.0)	0.442
Smoking status			0.302
Never smoker	6/9 (66.7)	62/83 (74.7)	
Ex-smoker	1/9 (11.1)	15/83 (18.1)	
current smoker	2/9 (22.2)	6/83 (7.2)	
Smoking amount (Pys)	30.0 (15.0, −)	30.0 (17.5, 44.0)	0.539
Initial symptom			
Dyspnea of exertion	6/9 (66.7)	58/71 (81.7)	0.373
Cough	6/9 (66.7)	49/72 (68.1)	1.000
Sputum	4/6 (66.7)	24/65 (36.9)	0.204
^*^Duration of symptom (month)	36.0 (3.5, 55.5)	3.0 (1.0, 6.0)	0.127
^*^CRP (mg/dL)	0.48 (0.31, 1.87)	0.45 (0.18, 1.31)	0.407
ANA (positive)	3/6 (50.0)	15/53 (28.3)	0.357
RF (positive)	1/4 (25.0)	10/55 (18.2)	0.572
Outcome			0.303
Alive	7/9 (77.8)	66/86 (76.7)	
Death	1/9 (11.1)	2/86 (2.3)	
Follow-up loss	1/9 (11.1)	18/86 (20.9)	

Note: Values in parentheses are percentages

*F:M* female:male, *FVC* forced vital capacity, *% pred* percentage of the predicted value, *FEV*_*1*_ forced expiratory volume, *DL*_*CO*_ diffusing capacity of the lung for carbon monoxide, *PaO*_*2*_ arterial oxygen tension, *CRP* C-reactive protein, *ANA* antinuclear antibody, *RF* rheumatoid factor

Note: data are expressed as the median with interquartile range or number with proportion (%)

^*^CRP showed non-normal distribution in the no treatment group

^*^Resting PaO_2_ mm Hg, duration of symptoms, and CRP showed a non-normal distribution in the treatment group

Table 3 also shows the difference in lung function between the initial PFT and 1-year follow-up PFT per the sero-positivity of ANA. The ANA results were available in 59 patients (62.1%). Forty-one patients (69.5%) with an initial negative ANA showed a significant improvement in lung function after 1 year; FVC (%) increased by 11.1%, FEV_1_ (%) by 11.3%, and DL_CO_ (%) by 12.1% (*p* = 0.008, *p* = 0.005, and p < 0.001, respectively). However, sero-positive patients did not show a significant improvement in lung function after 1-year.

**Table 3:** Comparison between initial and 1-year follow-up lung function according to antinuclear antibody (ANA) positivity

	ANA negative (*n* = 41)			ANA positive (*n* = 18)		
	Initial	Follow-up	p-value	Initial	Follow-up	*p*-value
FVC (%)	72.1 ± 20.6	83.2 ± 15.5	0.008	68.6 ± 19.5	76.4 ± 18.1	0.102
FEV_1_ (%)	80.1 ± 23.7	91.4 ± 19.7	0.005	75.4 ± 19.0	83.7 ± 19.1	0.091
DLco (%)	68.7 ± 24.9	80.8 ± 26.0	<0.001	62.9 ± 21.1	64.9 ± 23.4	0.568

*FVC* forced vital capacity, *% pred* percentage of the predicted value, *FEV*_*1*_ forced expiratory volume, *DL*_*CO*_ diffusing capacity of the lung for carbon monoxide, *ANA* antinuclear antibody

**Fig. 2:**
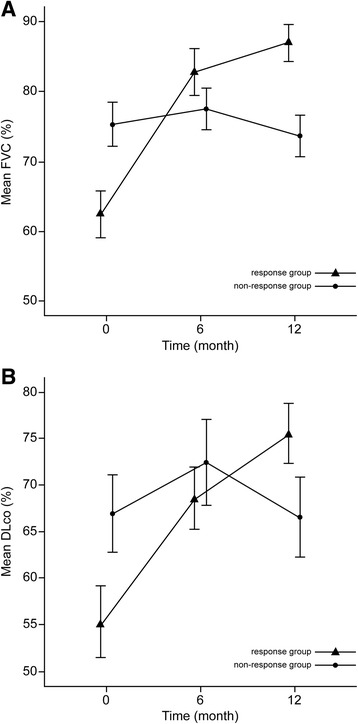
Changes in lung function over time between the response group and non-response group. **a** Change in functional vital capacity (FVC) (%) over time between the two groups (*p* < 0.001, Mean ± standard error (SE), **b** Change in the diffusing capacity of the lung for carbon monoxide (DL_CO_) (%) over time between the two groups (*p* = 0.002, Mean ± SE)

We compared the baseline characteristics between the response group and non-response groups (Table 4). Age and composition proportion of gender were similar between the groups (*p* = 0.895 and *p* = 0.705). In the response group, the follow-up duration was significantly longer than in the non-response group (*p* = 0.006), but the duration of respiratory symptoms was shorter (3.4 ± 4.4 months vs 7.1 ± 9.3 months, respectively; *p* = 0.038). With regard to pulmonary function, initial FVC (%), FEV_1_ (%), and DL_CO_ (%) were significant higher in the non-response group than in the response group (*p* < 0.001, *p* < 0.001, and *p* = 0.008, respectively). Current smokers were only found in the non-response group (*p* = 0.022). There were no significant differences in the initial respiratory symptoms, laboratory results, and comorbidities between the two groups (Table 4 and Additional file 1: Table S2). However, the proportion of ANA sero-positivity was higher in the non-response group than in the response group (*p* = 0.013). Furthermore, in the non-response group, two patients (4.5%) died during the follow-up period. Figure 2 shows the change in pulmonary lung function over time (initial, 6 month, and 12 month) between the two groups. The FVC improved by 24.6%, and DL_CO_ improved by 20.2% after 1 year in the response group. However, in the non-response group, lung function after 1 year did not differ greatly from baseline. Therefore, there were significant differences in FVC and DL_CO_ between the two groups over time (*p* < 0.001, and *p* = 0.002).

**Table 4:** Comparison of clinical characteristics between the response group and non-response group

	Response group (*n* = 42)	Non-response group (*n* = 44)	*p*-value
^*^Age (year)	55.5 ± 11.3	55.8 ± 9.7	0.895
F: M	28 (66.7): 14 (33.3)	31 (70.5): 13 (29.5)	0.705
Follow-up duration (month)	36.6 ± 14.1	28.6 ± 12.2	0.006
Pulmonary function test			
FVC (%)	63.6 ± 17.6	79.9 ± 18.4	<0.001
FEV_1_ (%)	71.7 ± 20.8	88.6 ± 19.9	<0.001
DLco (%)	56.9 ± 19.0	70.5 ± 25.0	0.008
Resting PaO_2_ mm Hg	81.4 ± 17.0	92.5 ± 11.0	0.004
Smoking status			0.022
Never smoker	30/40 (75.0)	32/43 (74.4)	
Ex-smoker	10/40 (25.0)	5/43 (11.6)	
current smoker	.	6/43 (14.0)	
Smoking amount (Pys)	26.5 ± 17.2	32.4 ± 15.1	0.415
Initial symptom			
Dyspnea of exertion	28/35 (80.0)	30/36 (83.3)	0.717
Cough	26/36 (72.2)	23/36 (63.9)	0.448
Sputum	13/33 (39.4)	11/32 (34.4)	0.675
^*^Duration of Symptom (month)	3.4 ± 4.4	7.1 ± 9.3	0.038
^*^CRP (mg/dL)	1.97 ± 5.01	1.19 ± 2.19	0.416
ANA (positive)	3/25 (12.0)	12/28 (42.9)	0.013
RF (positive)	2/25 (8.0)	8/30 (26.7)	0.092
Outcome			0.105
Alive	30/42 (71.4)	36/44 (81.8)	
Death	.	2/44 (4.5)	
Follow-up loss	12/42 (28.6)	6/44 (13.6)	

Note: Values in parentheses are percentages

*F:M* female:male, *FVC* forced vital capacity, *% pred* percentage of the predicted value, *FEV*_*1*_ forced expiratory volume, *DL*_*CO*_ diffusing capacity of the lung for carbon monoxide, *PaO*_*2*_ arterial oxygen tension, *CRP* C-reactive protein, *ANA* antinuclear antibody, *RF* rheumatoid factor

^*^Duration of symptoms, and CRP showed a non-normal distribution in the response group

^*^Age, duration of symptoms, and CRP showed a non-normal distribution in the non-response group

Multivariate analysis with logistic regression was conducted to investigate the risk factors for the non-response group (Table 5). Age, gender, duration of respiratory symptoms, FVC (%), DL_CO_ (%), and arterial oxygen tension (PaO_2_) were examined. Although ANA showed a significant difference between the response group and non-response group, only 15 patients showed positivity (Table 4). Thus, ANA was excluded from the multivariate analysis. The duration of symptoms at diagnosis was significantly associated with the response to treatment (hazard ratio (HR), 1.385; 95% CI, 1.058–1.813; *p* = 0.018). In addition, to identify the factors related to treatment failure, we defined patients with at least a 5% reduction in lung function reduction as “treatment failure” and performed logistic analysis. Thus, we found that age was significantly related to treatment failure. Older patients showed a tendency to experience treatment failure (Additional file 1: Table S4).

**Table 5:** Analysis of risk factors that affect treatment response by logistic regression

Variables	Odds ratio	95% CI	*p*-value
Age	1.026	0.913 to 1.152	0.665
Gender (M/F)	0.554	0.103 to 2.997	0.493
Duration of symptoms at diagnosis (Month)	1.385	1.058 to 1.813	0.018
FVC (% pred) at diagnosis	1.052	0.979 to 1.131	0.170
DL_CO_ (% pred) at diagnosis	0.995	0.951 to 1.042	0.838
PaO_2_ at diagnosis	1.014	0.954 to 1.079	0.645

*M/F* male/female, *FVC* forced vital capacity, *% pred* percentage of the predicted value, *DL*_*CO*_ diffusing capacity of the lung for carbon monoxide, *PaO*_*2*_ arterial oxygen tension, *CI* confidence interval

## Discussion

Although the prognosis of iNSIP is better than that of IPF, the 5-year mortality rate is estimated to be 17.7% [2]. To this date, there is no generally accepted guideline for the treatment of iNSIP; however, a previous study showed that corticosteroid and/or immunosuppressant therapy helped in maintaining or improving lung function in 81% patients with iNSIP [9]. Our study showed that the serologic negativity of ANA was related with an improvement in pulmonary function, and patients who had a relatively shorter duration of initial respiratory symptoms responded to corticosteroids better than iNSIP patients with a longer duration of initial respiratory symptoms.

Since Bjoraker et al. [13] reported the importance of the differentiation of NSIP from IPF, there has been much progression in the diagnosis of NSIP as a formally approved disease entity, but there are still no clear guidelines for diagnosis and treatment [2, 5, 12, 14]. Furthermore, some medical conditions (CTD-related ILD, hypersensitivity pneumonitis, cryptogenic organizing pneumonia, infection, or drug-induced lung disease) are related with NSIP, and therefore, the histologic characteristics of NSIP can be found in these diseases [15–18]. Due to the complexity of the diagnosis and low prevalence of NSIP, the factors predicting the response to treatment or the therapeutic effect are not well known [1, 6].

In the treatment group, lung function significantly improved after 1 year compared to the initial assessment; FVC (%) increased by 10.0%, FEV_1_ by 9.8%, and DL_CO_ by 8.4%. Park et al. [9] showed that the change in FVC (%) occurred according to histopathological type and the survival outcome; treatment response was better in cellular-type, and there was a 25% increase in FVC (%) in the cellular-type/survivor group after 1 year. However, there was no improvement in FVC (%) in the fibrotic-type/non-survivor group. Additionally, in their study, the initial mean FVC (%) was 63.6 ± 14.6, which was lower than what we observed. Xu et al. [8] also investigated the change in PFT results, but there was no significant improvement between the initial and follow-up lung function, possibly due to the non-fixed follow-up duration in their study. From our results, in the early stages of iNSIP, a clinician could anticipate that treatment would result in a 10% improvement in FVC (%) after 1 year. This information could help physicians predict the clinical course of patients with iNSIP, and plan adequate treatment modality.

Lee et al. [10] reported similar results to our study, showing that the presence of ANA was significantly related with disease progression and a poor response to corticosteroids. They suggested that sero-positivity of ANA could be an early manifestation of systemic diseases associated with a poor outcome of NSIP. Xu et al. [19] also showed that systemic autoimmune disease was significantly associated with increased mortality in NSIP patients (*p* = 0.023). Felicio et al. [20] reported a higher production of collagen and elastic fibers in NSIP with collagen vascular disease than in iNSIP; in a cohort of 41 NSIP patients, an increase in elastic fibers >1.5% was a significant risk factor for poor outcome (*p* = 0.01). In this study, the response group showed a lower proportion of ANA positivity than the non-response group (*p* = 0.013). Furthermore, negative ANA was associated with a significant improvement in lung function after 1 year.

Sawata et al. [21] studied the influence of smoking in 31 NSIP patients over 2 years. They showed that the smoking group had a significantly worse outcome than the non-smoking group; smoking was significantly related with a lower %DL_CO_/alveolar ventilation (DL_CO_/VA) in both iNSIP (*p* = 0.009) and CTD-NSIP (*p* = 0.044), and progression free survival was worse in the smoker group (*p* = 0.0489). Furthermore, they showed that exacerbation was common in a heavy smoker. Similarly, in our study, the non-response group had a relatively higher total smoking patient number than the response group, and current smokers were only observed in the non-response group (Table 4, *p* = 0.022). Marten et al. [22] suggested that emphysema is higher in smokers with NSIP; therefore, cigarette smoking could be a pathogenic factor in a subset of NSIP patients. Thus, we presumed that smoking is related with NSIP pathogenesis, and could provoke a poor response to corticosteroids, causing a worse outcome.

To assess the severity in the study population, we calculated the ILD-GAP score, which is a clinical prognosis prediction model using age, gender, and two lung function parameters (FVC (%), DL_CO_ (%)) [23, 24]. Although the initial PFT results were lower in the response group, the majority of patients in this study had ILD-GAP stage I (96.6%, data not shown). Moreover, a relatively longer duration of respiratory symptoms was a risk factor for poor response to corticosteroids (Table 5). This could mean that early treatment with corticosteroids and/or immunosuppressants might be more beneficial in the early stage of iNSIP, especially in ILD-GAP stage I patients. The physician should consider treatment of idiopathic NSIP in patients with respiratory symptoms, even if the severity of iNSIP is low.

There are some limitations to this study. First, it had a patient selection bias. This study was performed retrospectively and patients with ILD were enrolled in each hospital without a specific visit protocol. Therefore, 1-year follow-up PFT results exist in only 95 patients. Additionally, there have been major advances in the conceptualization of NSIP in recent years. In particular, it is currently speculated that iNSIP could be a type of autoimmune disease that is limited to the lungs or the respiratory manifestation of undifferentiated CTD [5, 18, 25]. Initially, we only enrolled patients without autoimmune disease and nine patients who developed CTD were excluded from this study. Nevertheless, there could be differences between this study population’s patients and currently diagnosed NSIP patients. Second, the NSIP subtype (cellular type, fibrotic type, or mixed) was not examined in this study. Previous studies showed that fibrotic NSIP was related with a poor prognosis and more frequent hospitalization [9, 11, 26]. If the subtype was investigated, it would be more informative. Third, the initial dose of corticosteroids or immunosuppressive agents was not examined. Lee et al. [10] reported that a low corticosteroid dose was significantly related with relapse, which could mean that the dose of corticosteroids administered could affect the response to treatment.

## Conclusion

Our study showed that corticosteroid and/or immunosuppressant therapy was effective in iNSIP, resulting in an improvement in lung function after 1 year. Corticosteroid-based treatment was especially effective in iNSIP patients who showed sero-negativity for ANA and those who had a shorter duration of respiratory symptoms. These findings suggest that early treatment with corticosteroids and/or immunosuppressants could be therapeutically beneficial in iNSIP patients, even if the disease is at an early stage. Further prospective, large, and well-designed studies are needed to confirm the factors affecting treatment effect.

## Additional file.

Additional file 1: Table S1. Treatment modality in treatment group (*n* = 86). **Table S2.** Comorbidities of study population. **Table S3.** Comparison between initial and 1-year follow-up lung function according to treatment. **Table S4.** Analysis of risk factors that associated with treatment failure (by logistic regression). (DOCX 26 kb)

## Abbreviations

% pred: percentage of the predicted value
ANA: anti-nuclear antibody
CI: confidence interval
CTD: connective tissue disease
DL_CO_: diffusing capacity of the lung for carbon monoxide
FEV_1_: forced expiratory volume
FVC: forced vital capacity
GAP: gender, age, and 2 lung physiology variables (FVC and DL_CO_)
HR: hazard ratio
ILD: interstitial lung disease
IPF: idiopathic pulmonary fibrosis
NSIP: nonspecific interstitial pneumonia
PaCO_2_: arterial carbon dioxide tension
PaO_2_: arterial oxygen tension
PFT: pulmonary function test
SD: standard deviation
SEM: standard error
TLC: total lung capacity
