# Supplementary material for: Factors affecting treatment outcome in patients with idiopathic nonspecific interstitial pneumonia: a nationwide cohort study
Source: Respir Res. 2017 Dec 6;18:204. doi: 10.1186/s12931-017-0686-7 (PMC5719588; doi:10.1186/s12931-017-0686-7)
Supplement: Additional file 1: Table S1. — Treatment modality in treatment group (n = 86). Table S2. Comorbidities of study population. Table S3. Comparison between initial and 1-year follow-up lung function according to treatment. Table S4. Analysis of risk factors that associated with treatment failure (by logistic regression). (DOCX 26 kb) [file 12931_2017_686_MOESM1_ESM.docx]

**Table S1**. Treatment modality in treatment group (n=86)

| Treatment modality | N (%) |
| --- | --- |
| Prednisolone | 47 (54.7) |
| Prednisolone + N-acetylcysteine | 4 (4.7) |
| Prednisolone + Azathioprine | 28 (32.6) |
| Prednisolone + Azathioprine + N-acetylcysteine | 2 (2.3) |
| Azathioprine | 4 (4.7) |
| Azathioprine + N-acetylcysteine | 1 (1.2) |

**Table S2.** Comorbidities of study population

|  | Total patients (n=261) | eligible patients (n=95) | Conservative care  group (n=9) | Treatment  group (n=86) | P-value | Response  group (n=42) | Non-response  group (n=44) | p-value |
| --- | --- | --- | --- | --- | --- | --- | --- | --- |
| Tuberculosis | 25 (9.7) | 13 (13.8) | 2 (22.2) | 11 (12.9) | 0.607 | 6 (14.6) | 5 (11.4) | 0.654 |
| Diabetes mellitus | 50 (19.2) | 14 (14.7) | 2 (22.2) | 12 (14.0) | 0.617 | 6 (14.3) | 6 (13.6) | 0.931 |
| Hypertension | 57 (21.8) | 26 (27.4) | 4 (44.4) | 22 (25.6) | 0.251 | 11 (26.2) | 11 (25.0) | 0.899 |
| Heart disease | 9 (3.4) | . | . | . | . | . | . |  |
| Cerebral disease | 3 (1.1) | 2 (2.1) | . | 2 (2.3) | 1.000 | 1 (2.4) | 1 (2.3) | 1 |
| Liver disease | 6 (2.3) | 2 (2.1) | . | 2 (2.3) | 1.000 | . | 2 (4.5) | 0.494 |
| Allergy | 4 (1.5) | 1 (1.1) | . | 1 (1.2) | 1.000 | 1 (2.4) | . | 0.488 |
| Renal disease | 2 (0.8) | 1 (1.1) | . | 1 (1.2) | 1.000 | . | 1 (2.3) | 1 |
| Chronic lung disease | 3 (1.1) | 1 (1.1) | . | 1 (1.2) | 1.000 | 1 (2.4) | . | 0.488 |
| Lung cancer | 3 (1.1) | 1 (1.1) | . | 1 (1.2) | 1.000 | 1 (2.4) | . | 0.488 |
| Other malignancy | 8 (3.1) | 2 (2.1) | 1 (1.1) | 1 (1.2) | 0.181 | . | 1 (2.3) | 0.488 |

Note: Values in parentheses are percentages.

**Table S3.** Comparison between initial and 1-year follow-up lung function according to treatment

|  | **No treatment group (n=9)** | | | **Treatment group (n=86)** | | |
| --- | --- | --- | --- | --- | --- | --- |
|  | Initial | Follow-up | p-value | Initial | Follow-up | p-value |
| FVC (%) | 78.3 ± 13.4 | 84.4 ± 12.9 | 0.276 | 71.9 ± 19.7 | 81.9 ± 17.1 | <0.001 |
| FEV_1_ (%) | 91.3 ± 16.8 | 95.6 ± 15.1 | 0.400 | 80.0 ± 21.8 | 89.8 ± 19.2 | <0.001 |
| DLco (%) | 72.4 ± 13.9 | 81.8 ± 40.2 | 0.489 | 65.0 ± 22.8 | 73.4 ± 21.9 | <0.001 |

**Table S4.** Analysis of risk factors that associated with treatment failure (by logistic regression).

| Variables | Odds ratio | 95% CI | p-value |
| --- | --- | --- | --- |
| Age | 1.164 | 1.000 to 1.355 | 0.049 |
| Gender (M/F) | 0.858 | 0.130 to 5.672 | 0.874 |
| Duration of symptoms at diagnosis (Month) | 1.042 | 0.941 to 1.155 | 0.427 |
| FVC (% pred) at diagnosis | 0.994 | 0.920 to 1.074 | 0.887 |
| DL_CO_ (% pred) at diagnosis | 0.976 | 0.919 to 1.036 | 0.418 |
| PaO_2_ at diagnosis | 1.091 | 0.995 to 1.196 | 0.065 |

Note: Treatment failure was defined as “at least a 5% reduction in lung function after treatment”.

M/F, Male/Female; FVC, forced vital capacity; % pred, percentage of the predicted value; DL_CO_, diffusing capacity of the lung for carbon monoxide; PaO_2_, arterial oxygen tension; CI, confidence interval.
